# Waiting time and its associated factors in patients presenting to outpatient departments at Public Hospitals of Jimma Zone, Southwest Ethiopia

**DOI:** 10.1186/s12913-022-07502-8

**Published:** 2022-01-25

**Authors:** Mensur Biya, Matebu Gezahagn, Bezawit Birhanu, Kiddus Yitbarek, Nigusu Getachew, Waju Beyene

**Affiliations:** 1 Catholic Organization for Relief and Development Aid, Jimma, Ethiopia; 2 Department of Health Policy and Management, Faculty of Public Health, Institute of Health, Jimma University, P.O. Box 378, Jimma, Ethiopia

**Keywords:** Waiting time, Associated factors, Public Hospital, Jimma zone, Ethiopia

## Abstract

**Background:**

Waiting time is defined as the total time that a patient spends in a facility from arrival at the registration desk until the time she/he leaves the facility or last service. In Ethiopia, the waiting time in the hospitals particularly in the outpatient department is lengthy. Studies at Jimma University specialized hospital indicated patients are forced to wait an average of 4.5 waiting hours to get service. Even there are many hospitals found in the zone, there is a paucity of information regarding waiting time and associated factors. Hence, this study is aimed to assess waiting time and associated factors at outpatient departments in Public Hospitals of Jimma zone, southwest Ethiopia.

**Methods:**

An institution-based cross-sectional study design was used from March 22 to June 3, 2020. A total of 422 study subjects were included in the study and systematic random sampling methods were used. The data were collected by observing the whole service points of each patient. The exit interview was made at the last point of the service unit. Descriptive statistics, bi-variable and multi-variable logistic regressions were used.

**Results:**

The whole waiting time patients spent in the hospitals before getting service was a minimum of 41 and a maximum of 185 min. Patients who came far from the hospitals were 1.93 times (AOR = 1.93; 95% CI, 1.16, 3.21) more likely to spend longer waiting time as compared to those who came from the hospital's area.

Patients visited on Monday were 2.64 times (AOR = 2.64; 95% CI, 1.45, 4.79) more likely to spend longer waiting time than those who visited the hospital on Friday. Patients who arrived early in the morning were 3.22 times (AOR = 3.22; 95% CI, 1.32, 7.86) more likely to spend longer waiting time than those who arrived in the afternoon.

**Conclusions:**

The mean waiting time was higher than the average recommended time by Business Process Reengineering (BPR) and more than five out of every ten clients spent long waiting time at outpatient departments Waiting time was affected by Educational status, residence, arrival time, and date of the visit.

## Background

Waiting time has been defined as the total time that a patient spends in a facility from arrival at the registration desk until the time she/he leaves the facility or last service. More specifically it is the length of time between enrolling a patient on a waiting list and the period that a patient takes at each point of service before being treated [1, 2].

It has been observed that long patient waiting times occur in both developed and developing countries, even though it may vary between countries, within a country, and from one health facility to another in the same geographic area [3].

Long waiting time adversely affects the willingness of the patient to return to the clinic which will highly reduce the utilization of health services [4]. Waiting time in hospitals is an important factor leading to patient dissatisfaction and making discomfort for the patient. A guideline of the American Institute of Medicine states that patients must be seen within 30 min after they arrive at a hospital [5].

Excessive waiting time is a lose-lose strategy in that patients lose their valuable time; hospitals lose their patients and staff experience tension and stress [6]. In many health care systems throughout the world, it is common of being postponed to access medical services [7]. This long waiting time is a worldwide phenomenon that needs much more to be done to reduce patient waiting time in public hospitals [6].

Usually, it is observed that patients at the hospital OPD have to wait for a disproportionately long time before they can get medical treatment or advice from professional healthcare workers. In a competitively managed healthcare environment, the long waiting time of patients in an OPD adversely affects the hospital's ability to attract new increased business [8].

Studies in most developing countries show that patients spend 2 to 4 h in the outpatient departments before seeing the doctor. A study carried out at the outpatient departments in the holy family hospital, Tuchman, Ghana found out that the mean waiting time at records was the highest, 74.5 min [9]. In Ethiopia, the waiting time in the hospitals particularly in the outpatient department is lengthy. Studies at Jimma University specialized hospital indicated patients are forced to wait an average of 4.5 waiting hours to get service [10]. Even if there are many hospitals found in the zone there is a paucity of information regarding waiting time and if the information presented it was not complete and analyzed to make a decision Studying this concept is highly important to improve the quality of the care in terms of client satisfaction, Therefore this study fills the gap by assessing waiting time and identifying the potential factors that contribute to long waiting time at the outpatient department in public Hospitals of Jimma zone, southwest Ethiopia.

## Methods

### Study design, setting and period

A facility based cross sectional study design was conducted in Jimma Zone Hospitals southwest of Ethiopia from March 22 to June 3, 2020. The zone has eight governmental hospitals, two private hospitals, and 120 health centers. Out of the eight governmental hospitals, one is a referral hospital Jimma University Medical Center (JUMC), three are general hospitals and four are primary hospitals. It provides service for a total population of 2,488,155 out of which 1,255,527 are male and according to 2019 hospitals report the outpatient follow was 32,000 adults (Plan, and program office).

### Population

The source and study population were all and randomly selected patients who visited the out-patient department in the hospitals respectively. All clients ≥ 18 years and who visited the outpatient department during the data collection period were included in the study and Clients who were severely ill and who came for follow up and repeated procedure were excluded in the study.

### Sample Size Determination and sampling procedure

**Fig. 1:**
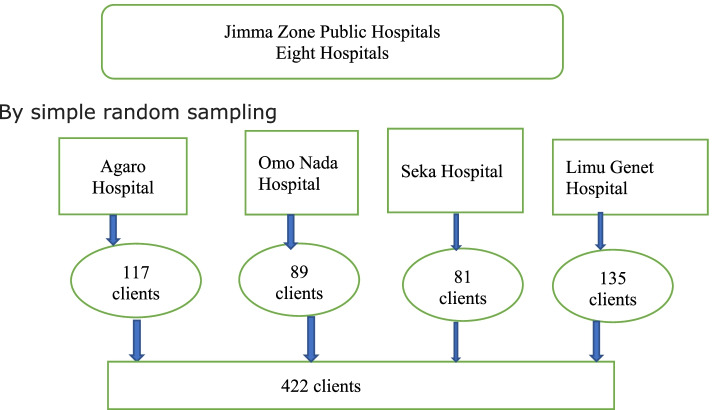
Schematic presentation of sampling procedure

The single population formula was used assuming 95% confidence interval and 50% prevalence (P) due to lack of such study, and a precision of 5% between the sample and the 10% non-response rate is taken, thus a total of 422 clients required for the study. Simple random sampling techniques were used to select the hospitals. From the total of eight hospitals found in the Jimma zone, four hospitals (Agaro general hospital, Omo Nada primary hospital, Seka primary hospital, and Limu Genet general hospital) were selected for the study and after proportional allocation to each hospital based on the outpatients flow, 117clients from Agaro hospital, 89 clients from, Omo Nada primary hospital 81 clients from Seka, and 135 clients from Limmu genet hospital were selected by systematic random sampling method and the first sample was selected randomly by lottery method and data was collected from each of the fifth client (k = 5) in each of the hospitals till the allocated sample size reach (see Fig. 1).

### Study variables

The dependent variable was waiting time and the independent variables were socio-demographic variables and health Service related variables date of visit, arrival time, the purpose of visit, previous health facility visited Frequency of visit, and reason for failure to visit another facility.

### Operational Definitions

**Waiting time** was the time a patient had to wait at registration, consultation, laboratory, and other diagnostic units and at the pharmacy to receive service.

**Overall waiting time** was the addition of waiting time at each service.

- If the cumulative patient waiting time was greater or equal to 120 min considered as long waiting time and
- If the cumulative waiting time is less than 120 min it is considered as short waiting time and Business Process Re-engineering (BPR) calculated a maximum of 2 h waiting time for hospital OPD.

**Arrival time:** the time the patient reaches the registration room and it is classified in to the following categories.

**Early in the morning**: The patient reached the hospital's registration room with in the time interval of 8:00 am to 11:00 am.

**Late morning**: The patient reached the registration room in the time between 11:00 am to 12:00 pm.

**Mid-day**: The time between 12:00 pm and 1:00 pm.

**Afternoon**: Includes the time of a day from 1:00 pm to 5:00 pm.

**Departure time:** This is the moment the client or the patient exit the hospital.

### Data collection instrument and procedure

Data was collected by Four BSc nurses and two supervisors using both independent observation and interview starting from (2:30 am to 5:30 pm) on five working days for 51 days. The data collectors collected the data by observing the whole service points of each patient. The exit interview was made at the last point of the service unit. The tools were adapted by reviewing different kinds of literature [1, 5, 11, 12]. The first tool was the time and motion tool that measures time by using independent observation for each patient in each unit of service delivery. This tool was used to track the patient flow from the time they entered, through various service stations until the time they exit from the hospital using an adjusted watch. The second tool was an interviewer-administered translated structured questionnaire. This tool assesses various factors that are associated with waiting time. The tool had two sections; questions assessing the socio-demographic characteristics of patients and patient-related clinical factors.

### Data processing and analysis

After the completion of data collection, data were checked for completeness, edited, cleaned, coded, and entered into Epi-Data version 3.1 and exported to SPSS version 20 for analysis. The presence of Multi-co linearity was checked for independent variables using the Variance inflation factor and no variable is multi-co linearity with a maximum VIF of 1.03. Overall waiting time was transformed to dichotomous for bi-variable analysis. In the bi-variable logistic regression analysis p-value, less than 0.25 was used to select the candidate variables for multivariable logistic regression analysis. The final model was made using a Forward LR method. P-values less than 0.05 were used as cut-off-point to determine association in the final multivariable logistic regression analysis model. The goodness of fit of the models was checked by the Hosmer–Lemeshow goodness. Finally, descriptive statistics, text narration, and tables were used to present the results.

### Ethics approval and consent to participate

Ethical clearance was taken from the Institutional Review Board of Jimma University Institute of health, and a permission letter was obtained from the zonal health department including from the respective hospital management. Written informed consent was obtained from all study participants before the data collection. Name and other personal identifiers were not recorded to maintain confidentiality. All the protocol was performed following the relevant guideline and regulation. While approaching the respondents, necessary personal protective measures (wearing masks, using sanitizer, and keeping social distancing) were taken to reduce the risk of transmission of COVID19.

## Results

### Socio-demographic characteristics

A total of 423(100%) clients were included in the exit interview and Majority 408(96.5%) of the respondents participated in the study. Among them, 222 (54.4%) were males, the age of the highest proportion 186 (45.6%) of the respondents lies between twenty and thirty years with an average age of 33.4 and standard deviation of ± 10.7. More than two-thirds of the study participants 274 (67.2%) were married. Regarding educational and occupational status, 98(24%) of the participants were unable to read and write and 124(30.4%) were farmers respectively (See Table 1).

**Table 1:** Socio-Demographic Characteristics of the Respondent at OPD in public Hospitals of Jimma Zone Southwest, Ethiopia, 2020

Variable	Frequency	Percentage
**Sex**		
Male	222	54.4
Female	186	45.6
**Age (in a year)**		
<—19	12	2.9
20—30	186	45.6
31—40	81	19.9
41—50	91	22.3
51—60	25	6.1
61 +	13	3.2
**Marital status**		
Single	106	26
Married	274	67.2
Divorced	15	3.7
Widowed	13	3.2
**Educational status**		
Illiterate	98	24.0
Read & write	62	15.2
Primary	62	15.2
Secondary	31	7.6
Preparatory	93	22.8
Tertiary	62	15.2
**Employment status**		
Student	62	15.2
Merchant	58	14.2
Farmer	124	30.4
Civil servant	49	12.0
NGO	9	2.2
**Residence**		
Hospitals Area	307	75.2
Out of Hospitals Area	101	24.8

### Service-related characteristics

One hundred thirty-seven (33.6%) of the respondents visited the hospital on Monday and one hundred twenty-nine (31.6%) arrived early in the morning. Before the respondents came to the hospital outpatient departments, 175 (42.9%) had visited other health facilities for a similar reason, 79 (52%) had visited health centers, About 94 (36.7%) of the respondents came to the hospital looking for a better service. Two hundred thirty-one (56.6%) of the respondents have visited the hospital more than once and 182 (44.9%) were the self-referred client (See Table 2).

**Table 2:** Patient-Related Characteristics of the Respondent at OPD in public Hospitals of Jimma Zone Southwest, Ethiopia, 2020

Variable	Frequency	Percent
**Date of visit**		
Monday	137	33.6
Tuesday	62	15.2
Wednesday	81	19.9
Thursday	42	10.3
Friday	86	21.0
**Arrival time**		
Early morning	185	45.3
Late morning	111	27.2
mid-day	84	20.6
afternoon	28	6.9
**frequency of visit**		
new attend	170	41.7
repeat attend	238	58.3
**Purpose of visit**		
Appointment	91	22.3
Referred	91	22.3
Self-refer	226	55.4
**Visited another health facility**		
Yes	175	42.9
No	233	57.1
**Previous facilities visited**		
Health center	89	51
Drug shop	45	25.7
Private clinic	30	17.1
Traditional healer	11	6.2
**Reason for failure to visit another** facility		
Short distance	65	27.9
Cost	77	33
Better service	91	39.1

### Waiting time of the patients at different departments

The minimum and maximum waiting time at registration was 6 and 30 min respectively with a median of 18 min (± 4.9) minutes. From 408 study participants, 244 (59.8%) were sent to the laboratory for investigation and 356 (87.3%) patients had a prescription to the pharmacy. The waiting time at laboratory service was a minimum of 11 and a maximum of 92 min with a median of 31 min (± 20.7 min). Forty-four (10.8%) patients were sent to other diagnostic (X-ray and ultrasound) units their waiting time ranges from 21 to 52 min with a median of 33 min and a standard deviation of ± 10.3 min (See Table 3).

**Table 3:** Waiting Time at Each Service Unit of the OPD in public Hospitals of Jimma Zone Southwest, Ethiopia, 2020

**Unit**	**Number of clients attended**	**Mean waiting time**	**Standard deviation**	**Minimum**	**Maximum**
**Registration**	408	18	± 4.9	6	30
**Examination**	408	21	± 21.2	4	78
**Laboratory**	244	31	± 20.7	11	92
**Other diagnostics**	44	33	± 10.3	21	52
**Pharmacy**	356	16	± 6.1	6	33

### The overall waiting time of the patients

**Fig. 2:**
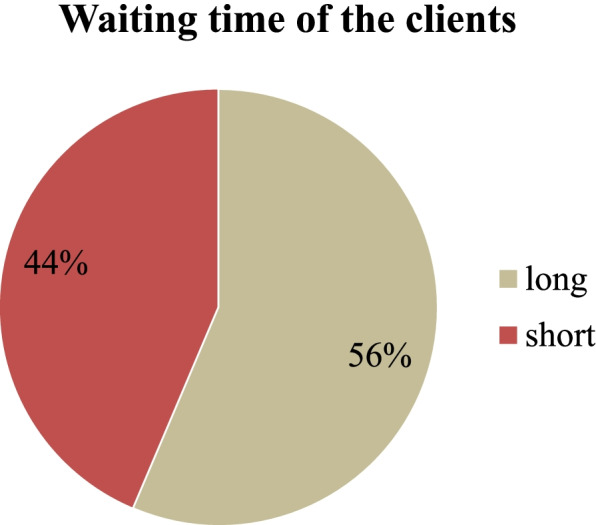
The overall waiting time respondent spent at OPD in public Hospitals of Jimma Zone Southwest, Ethiopia, 2020

The minimum and the maximum waiting time of patients were 41 min to 185 min respectively with a median of 93 min ± 33.4 min The commutative patient waiting time was greater or equal to 120 min considered as long waiting time and less than 120 min considered as short waiting time as defined operationally above. Of the total participants, 56.4% have spent long waiting times (See Fig. 2)**.**

### Waiting time with different service-related characteristics

**Fig. 3:**
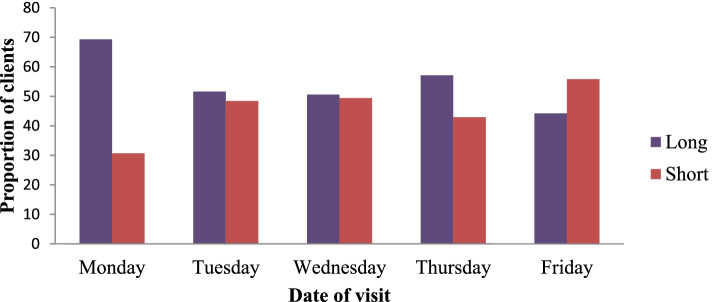
Waiting time with the date of visit at OPD in public Hospitals of Jimma Zone Southwest, Ethiopia, 2020

One hundred thirty-seven (33.6%) of the respondents visited the hospital on Monday and 129 (31.6%) arrived early in the morning. Of the respondents who visited the hospital on Monday, 69.3% spent a longer waiting time and of the respondents who arrived early in the morning, 64.3% spent longer waiting time (See Fig. 3).

**Fig. 4:**
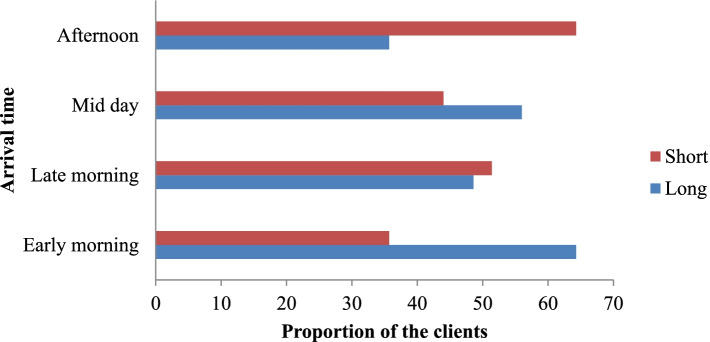
Waiting time of the respondent with their arrival time in public Hospitals of Jimma Zone Southwest, Ethiopia, 2020

The majority of the patients who arrived early in the morning were waited for a long period to get the service in the hospital. On the contrary majority of clients who were arrived in the afternoon were waited for a short time (See Fig. 4).

### Factors associated with waiting time of the clients

All candidate variables were entered into a multivariable logistic regression to control potential confounding factors. In multivariable logistic regression, educational status, residence, arrival time and date of the visit were significant at *a p*-value of < 0.05.

A patient who was unable to read and write was 2.25 times (AOR = 2.25; 95% CI, 1.11, 4.58) more likely to spend longer waiting time at a hospital facility as compared to those with tertiary education. Patients who came far from the hospitals were 1.93 times (AOR = 1.93; 95% CI, 1.16, 3.21) more likely to spend longer waiting time as compared to those who came from the hospital's area.

Patients who visited the hospital on Monday were 2.64 times (AOR = 2.64; 95% CI, 1.45, 4.79) more likely to spend longer waiting time at the hospital facility as compared to those who visited the hospital on Friday. Regarding arrival time those who came early in the morning were 3.22 times (AOR = 3.22; 95% CI, 1.32, 7.86) more likely to spend longer waiting time than those who arrive in the afternoon (See Table 4).

**Table 4:** Multivariable analysis of waiting time and associated factors at OPD in public Hospitals of Jimma Zone Southwest, Ethiopia, 2020

**Variable**		Waiting time		**COR (95% CI COR)**	**AOR (95%CI AOR)**	***P*-value**
		**Long Waiting time N (%)**	**Short Waiting time N (%)**			
**Educational status**	Illiterate	69(74.2)	24(25.8)	2.87(1.45, 5.68)	2.25(1.11, 4.58)*	0.025
	Read & write	29(46.8)	33(53.2)	0.87(0.43, 1.77)	0.84(0.40, 1.76)	0.643
	Primary	30(48.4)	32(51.6)	0.94(0.46, 1.89)	0.73(0.35, 1.53)	0.405
	Secondary	15(48.4)	16(51.6)	0.94(0.39, 2.22)	0.83(0.34, 2.05)	0.689
	Preparatory	56(57.1)	42(42.9)	1.33(0.71, 2.52)	1.05(0.53,2.01)	0.883
	Tertiary	31(50)	31(50)	1	1	
**Residence**	Out of Seka	69(68.3)	32(31.7)	1.95(1.22, 3.15)	1.93(1.16, 3.21)*	0.011
	Seka	161(52.4)	146(47.6)	1	1	
**Date of visit**	Monday	95(69.3)	42(30.7)	2.85(1.63, 4.99)	2.64(1.45, 4.79)*	0.001
	Tuesday	32(51.6)	30(48.4)	1.35(0.70, 2.59)	1.34(0.67, 2.71)	0.409
	Wednesday	41(50.6)	40(49.4)	1.29(0.71, 2.38)	1.27(0.67, 2.42)	0.455
	Thursday	24(57.1)	18(42.9)	1.68(0.80, 3.55)	1.42(0.65, 3.12)	0.384
	Friday	38(44.2)	48(55.8)	1	1	
**Arrival time**	Early morning	119(64.3)	66(35.7)	3.25(1.46, 7.43)	3.22(1.32, 7.86)*	0.010
	Late morning	54(48.6)	57(51.4)	1.71(0.72, 4.02)	1.75(0.69, 4.38)	0.232
	Mid-day	47(56.0)	37(44.0)	2.28(0.94, 5.54)	2.24(0.87, 5.74)	0.092
	Afternoon	10(35.7)	18(64.3)	1	1	

^*^ *P* ≤ 0.05 *AOR* adjusted odds ratio*, CI* confidence interval

## Discussion

While it is well established that shorter wait times are positively associated with clinical provider scores of patient satisfaction, results indicated that every aspect of patient experience-specifically confidence in the care provider and perceived quality of care-correlated positively with shorter wait times.

This study showed that the maximum waiting time at registration was 30 min. It is higher than the standard stated waiting time of 7 min given by the Business Process Reengineering (BPR) program [13]. It could be due to in the study area there, may be a large number of patients in the queue and the long searching of cards. While it is lower compared to a study done in Ghana Holy Family Hospital that was 2 h and 15 min were waiting for registration [9]. This difference might be due to socio-demographic varieties and the difference in setting and level of hospitals. Another study done at primary care facilities in Trinidad and Tobago revealed that the average waiting time in hospitals is 2 h and 40 min, with a range of less than 1 h to 6 h which is longer than the current study [14].The possible reason might be due to difference in the study areas and level of the hospitals.

In this study, the average waiting time for consultation was 31 min. This finding was consistent with a study done in Hosanna Outpatient Department that was 30.9 min [15]. And it is lower while compared to Tertiary Care Hospital in Pune that was 40 min [16]. This difference might be due to the setup and the level of the hospitals.

Findings from this study showed that the mean waiting time at the laboratory was 39.8 min. This finding is lower than the study done in Jimma Medical Center that was 68.6 min [17].This variation could be a difference in the study setting that the current study took place at a primary hospital, and some investigations may not be present in the hospitals.

This study found that the mean total waiting time was 93 min. This finding is lower when compared to Felege Hiwot Hospital, Bahir Dar, in which was the average waiting time was149 minutes [18]. And also lower when compared to a study conducted at Jimma Medical Center that was an average total waiting time of 4.5 waiting hours [19]. This discrepancy might be because of the difference in the level of facilities in measurement, data collection method, and the associated size of the catchment population. Both Felege Hiwot and Jimma Medical centers were teaching hospitals serving over five million populations in their catchment. However, the other hospitals were only providing service to the people of the catchment population of the hospitals.

In current study patients who was unable to read and write was 2.25 times more likely to spend longer waiting time at hospital as compared to those with tertiary education. This is nearly similar with the study conducted in OPD of Mulago Hospital, Uganda, in which the mean overall waiting time of a patient who had ever attained tertiary education was 79.1 min less than patients who had never attained any formal education [1].

Regarding arrival time those who came early in the morning were 3.22 times more likely to spend longer waiting time than those who arrive in the afternoon. This is comparable to the study conducted at OPD of Nairobi health service shows a patient who arrives late morning takes the longest time 59.1 min and a Patient who arrives in the afternoon takes a shorter time 48.6 min [5].

### Limitation of the study

- There might be Social desirability bias (During the exit interview the clients may show courtesy bias).
- Other the study was not involving all departments and it lacks qualitative parts.
- Since the study used cross sectional design, does not determine the cause and effect over a period of time.

## Conclusion

The median outpatient waiting time was long and more than five out of every ten clients spent long waiting time at the hospitals OPD. The study also elucidates important factors that affect and determine the overall waiting time. Educational status, residence, arrival time, and date of the visit were statistically associated with waiting time. Hence, all concerned bodies to consider and maintain factors identified in this study in their service provision practice to foster a higher level of quality of service in terms of client satisfaction with a minimal waiting time in the hospitals.

## Data Availability

Data will be available upon request from the corresponding author.

## Abbreviations

ANC: Antenatal care
CVD: Cardio Vascular Disease
IOM: Institute of Medicine
LoF: Length of stay
NHS: National Health Service
OECD: Organization for Economic Co-operation and Development
